# Comprehensive Physiotherapy Approach for Pneumonia After Angioplasty in an 83-Year-Old Hypertensive Male Patient: A Case Report

**DOI:** 10.7759/cureus.55454

**Published:** 2024-03-03

**Authors:** Sojwal P Nandanwar, Lajwanti Lalwani, Priyanka K Chilhate

**Affiliations:** 1 Department of Cardiovascular and Respiratory Physiotherapy, Ravi Nair Physiotherapy College, Datta Meghe Institute of Higher Education and Research, Wardha, IND

**Keywords:** outcome measures, relaxation techniques, pneumonia, physiotherapy intervention, coronary artery disease, angioplasty

## Abstract

Pneumonia is an infection that causes inflammation in the air sacs of the lungs. Coronary artery disease is a condition characterized by the buildup of plaque in the coronary arteries, which supply blood to the heart. This obstruction restricts blood flow, resulting in chest pain (angina) and, in extreme cases, heart attacks. An important part of successfully treating diseases like peripheral artery disease and coronary artery disease is balloon angioplasty, a commonly used medical procedure for treating narrowed or clogged arteries. An 83-year-old man who had pneumonia after angioplasty was the subject of this case study. The patient had pneumonia after angioplasty, which was managed by proper medications and cardio-respiratory physiotherapy. The patient was intubated and referred for cardio-respiratory physiotherapy. Physiotherapy treatments like mild chest vibrations, suctioning, and bed mobility exercises were given initially. After extubation, physiotherapy treatment continued with deep breathing exercises, coughing techniques, relaxation techniques, and mobility exercises for the upper limbs and lower limbs. Effective physical rehabilitation was necessary in order to minimize complications following angioplasty and allow him to resume his daily activities. Several outcome measures, like the ICU mobility scale, CURB-65 score, and chest X-ray grading scores, were used to monitor the patient's progress during rehabilitation. The benefits of pulmonary rehabilitation programs emphasize the need for tailored approaches in addressing individual patient needs for comprehensive recovery.

## Introduction

Pneumonia is a common acute respiratory infection that affects the lung's alveoli and distant airways. It can cause serious illness and death in all age groups, both in the short and long term. The inflammation of one or both lungs, frequently brought on by bacterial, viral, or fungal invaders, is what distinguishes it. Community-acquired pneumonia and hospital-acquired pneumonia, which include ventilation-associated pneumonia, are the two main categories into which the illness falls [1]. As a subtype of ICU-acquired pneumonia, ventilator-associated pneumonia (VAP) is characterized by infection of the pulmonary parenchyma in patients subjected to invasive mechanical ventilation for at least 48 hours. When a patient needs invasive mechanical ventilation, one of the most frequent infections is still VAP. Bacteria, viruses, or fungi can bring on lung infections such as pneumonia. It is characterized by inflammation of the air sacs in the lungs, causing symptoms like coughing, breathing problems, and chest pain [2].

Coronary artery disease (CAD) is an artery disease where a buildup of cholesterol and other fats causes the coronary arteries to narrow or become obstructed. This may impede the heart's ability to receive blood, which could lead to potentially fatal consequences like heart attacks and chest pain [3]. When community-acquired pneumonia was present, cardiovascular problems affected 2.1% of outpatients and 26.7% of inpatients [4].

Balloon angioplasty, a widely used medical technique for treating constricted or obstructed arteries, is critical in effectively managing conditions such as coronary artery disease and peripheral artery disease. When performing this procedure on older adults, while performing this procedure in the elderly population that is 65 years old and above, it is critical to carefully assess their unique health needs and potential obstacles [5]. Balloon angioplasty in geriatric patients necessitates a cautious and personalized approach. Because of the high prevalence of other health conditions in this population, it is critical to conduct a thorough assessment of conditions such as diabetes, hypertension, and renal impairment prior to the procedure in order to manage them effectively. The selection of an appropriate site for vascular access, often using the radial or femoral approach, is critical because it accounts for potential difficulties related to vessel size and calcification. Proper medication management, including anticoagulant and anti-platelet therapy adjustments, is critical in balancing the risk of bleeding and clotting [6].

Cardio-respiratory physiotherapy is a method that aims to improve a patient's respiratory status and hasten their recovery by teaching cough and breathing techniques, using manual vibrations on the chest wall, improving airway clearance in lung diseases associated with reduced airway resistance, and improving patient positioning [7]. Though its application of manual chest clearance techniques varies, evidence-based practice consistently encourages early mobilization [8]. The physiotherapy protocol included deep breathing exercises, incentive spirometry, and mobilization techniques and exercises. It has been demonstrated that the recommended exercises increase strength, mobility, range of motion, and fitness [9].

We are presenting a case of an 83-year-old hypertensive male who has undergone angioplasty, requiring efficient physical rehabilitation to speed up recovery by preventing or resolving post-respiratory and cardiovascular complications and providing physical rehabilitation to restore their functional ability. Key outcome measures include the ICU mobility scale, CURB-65 score, and chest X-ray grading scores.

## Case presentation

An 83-year-old male hypertensive patient resident of Sawangi visited the emergency care department with a complaint of cough with whitish expectoration, shortness of breath which was slow onset and continuous throughout the day, continuous low-grade fever, and chest pain for 15 days. He also gave a history of identical concerns experienced 17 years ago on 21st May 2006; he visited a government hospital (Nagpur), where he was suggested diagnostic tests like 2D-echo and color Doppler and diagnosed as left anterior descending artery blockage and managed conservatively. The patient experienced the same symptoms on 14th October, for which he was suggested diagnostic tests like 2D-echocardiogram, radiography, arterial blood gas, and routing tests (liver function test, kidney function test, and complete blood count). 2D-echocardiogram revealed that the left ventricular ejection fraction is 60% and grade I mild concentric left ventricular mass. Thorax radiography showed homogeneous opacity on the left middle and lower lung fields. Arterial blood gas showed fully compensated respiratory alkalosis. He underwent balloon angioplasty of the left anterior descending artery on 15th October 2023. He shifted to the medicine intensive care unit and was diagnosed with pneumonia and referred for cardio-respiratory physiotherapy. Timeline with events mentioned in Table 1.

**Table 1:** Timeline with events.

Sr. no	Events	Date of events	Description of events
1	Date of previous hospitalization	21-May-2006	Cough with whitish expectoration, shortness of breath
2	Date of emergency admission	14-October-2023	Cough with whitish expectoration, shortness of breath, low-grade fever and chest pain
3	Date of pre-surgery physiotherapy evaluation	14-October-2023	Patient and family education
4	Date of surgery	15-October-2023	Balloon angioplasty
5	Date of intubation and post-angioplasty physiotherapy evaluation	16-October-2023	Mild vibrations and suctioning, bed mobility exercises
6	After extubation, continue with cardio-respiratory physiotherapy treatment.	19-October-2023	Deep breathing exercises, coughing techniques, and mobility exercises for the upper limbs and lower limbs.

Clinical finding

For clinical examination, patient consent was taken. Clinical findings are mentioned in Table 2.

**Table 2:** Post-operative clinical finding. BiPAP: bilevel positive airway pressure, CPAP: continuous positive airway pressure, PEEP: positive end-expiratory pressure, mmHg: millimeter(s) of mercury, cm: centimeter.

Day	Patient position and O_2_ support	Clinical finding	Inspection and examination
Day one	The patient was examined in a supine-lying position. He was on mechanical ventilator BiPAP mode with FiO_2_-25% and PEEP-6 cm H_2_O via an endotracheal tube.	Heart rate: 98 beats per minute, blood pressure: 136/94 mmHg, respiratory rate: 22 breaths per minute, saturation of oxygen: 99%	On inspection, Ryle’s tube, Foley’s catheter, and IV line. On auscultation, crackles were present bilaterally in the middle and lower zones, and breathing sounds were reduced on the left side.
Day two	The patient was examined in a supine-lying position. The patient was on bubble CPAP via face mask.	Heart rate: 88 beats per minute, blood pressure: 128/86 mmHg, respiratory rate: 18 breaths per minute, saturation of oxygen: 98%	On inspection, Ryle's tube, Foley's catheter, and IV line. On auscultation, crackles were present bilaterally in the middle and lower zones, and breathing sounds were reduced on the left side.
Day four	The patient was seen in half lying. He shifted on four liters of oxygen via nasal prongs.	Heart rate: 82 beats per minute, blood pressure: 126/88 mmHg, respiratory rate: 16 breaths per minute, saturation of oxygen: 99%	On inspection, the IV line and Foley's catheter. On auscultation, crackles were presented over bilateral lower zones.
Day eight	The patient was seen in a sitting position. He was on room ventilation.	Heart rate: 84 beats per minute, blood pressure: 112/74 mmHg, respiratory rate: 22 breaths per minute, saturation of oxygen: 99%	On auscultation, crepitus was present in the middle zones, and air entry bilaterally was reduced. On assessment, chest expansion at the level of the axillary, nipple, and xiphoid processes revealed differences of 2 cm, 2cm, and 3 cm, respectively.
Day 14	The patient was seen sitting; he was on room ventilation.	Heart rate: 88 beats per minute, blood pressure: 112/74 mmHg, respiratory rate: 22 breaths per minute, saturation of oxygen: 99%	On auscultation, air entry was bilaterally reduced. On assessment, chest expansion at the level of the axillary, nipple, and xiphoid processes revealed differences of 2 cm, 3 cm, and 4 cm, respectively.

Diagnostic assessment

**Figure 1:**
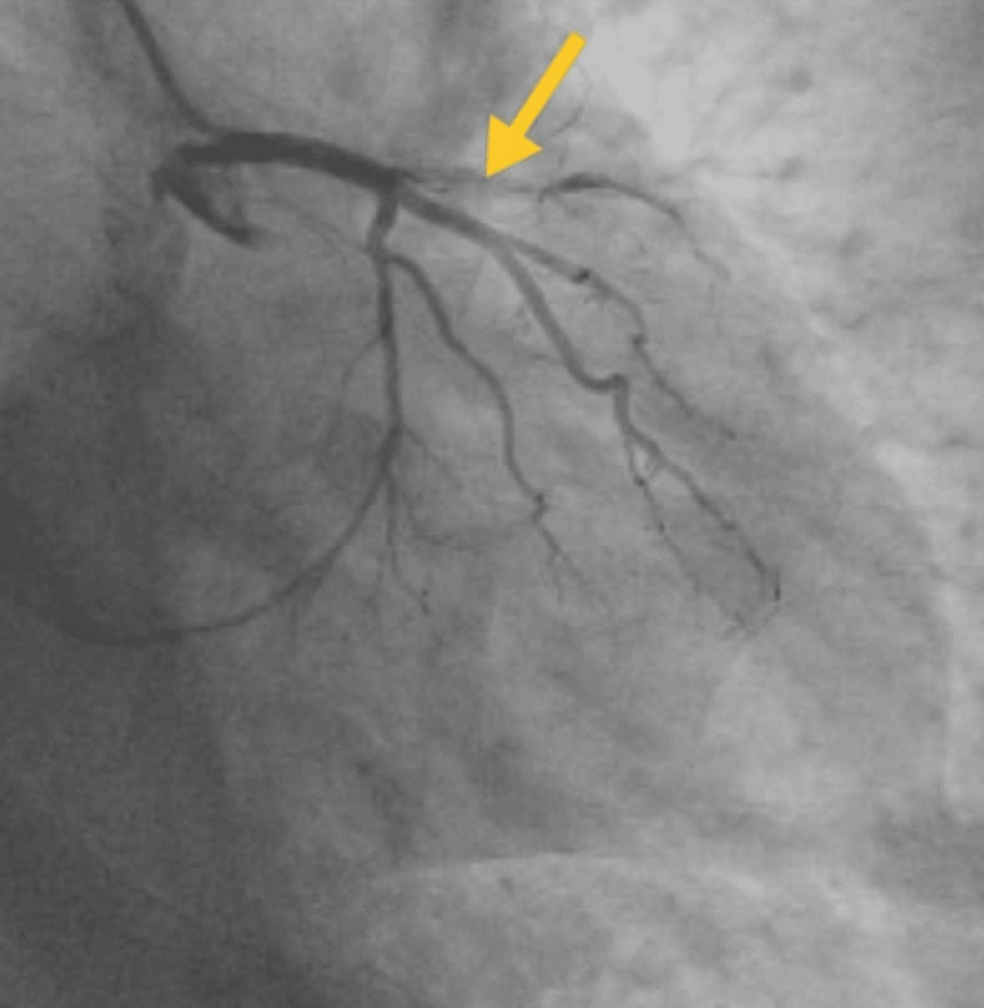
Coronary angiography findings. The yellow arrow shows the left anterior descending coronary artery blockage.

**Figure 2:**
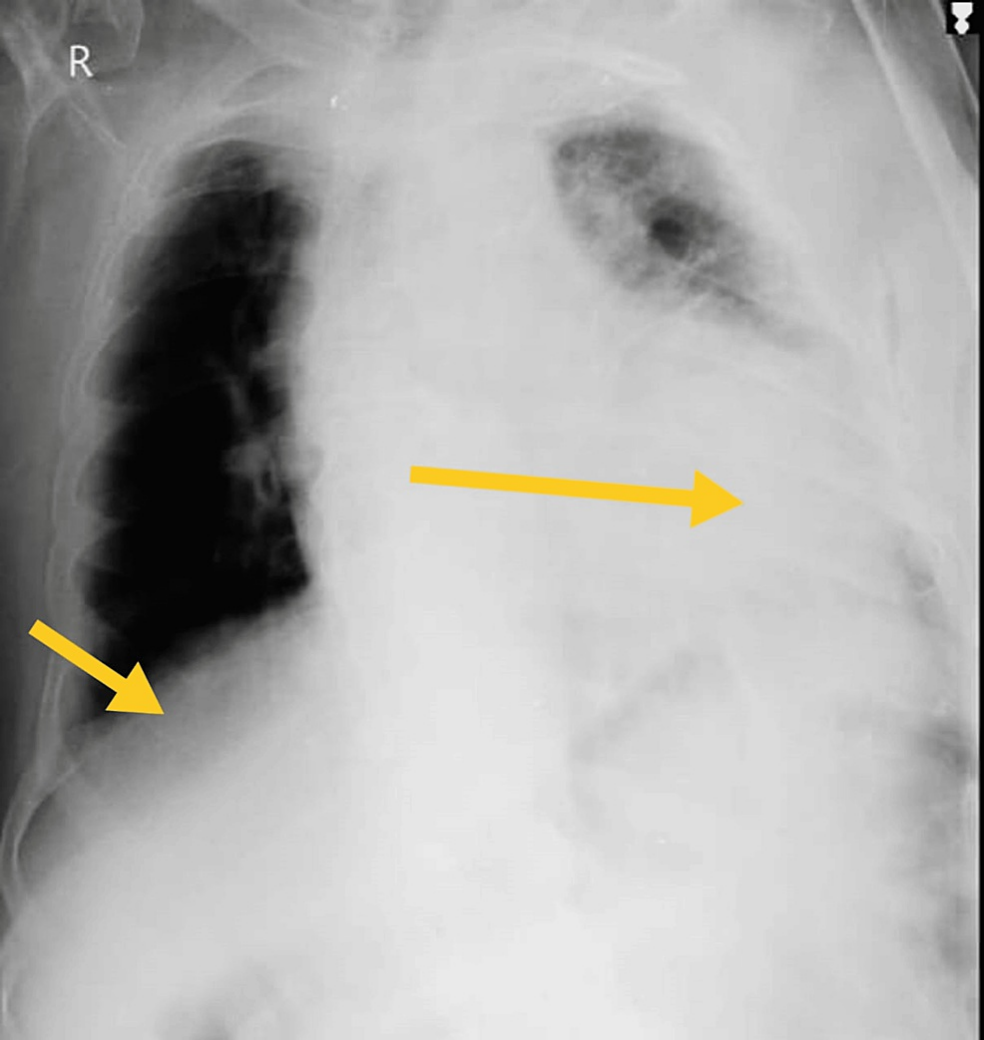
Thoracic radiography on 14th October 2023. The yellow arrow shows homogeneous opacity in the left middle lung field, the lower lung field, and the right lower lung field.

2D-echocardiogram revealed that the left ventricular ejection fraction is 60% and grade I mild concentric left ventricular mass. Coronary angiography revealed left anterior descending coronary artery blockage is shown in Figure 1. Thorax radiography showed homogeneous opacity on the left middle and lower lung field. The thorax radiological findings are mentioned in Figure 2. Complete blood count reduced hemoglobin (11%), and the total erythrocyte count was 3.75 million cells per microliter. Kidney function test-creatinine: 2.1 milligrams/deciliter; liver function test-alanine aminotransferase: 152 international units per liter. Random blood glucose: 166 milligrams/deciliter. Arterial blood gas finding: on day one, the arterial blood gas report showed fully compensated respiratory alkalosis, and after week two, the arterial blood gas report showed normal findings.

Post-angioplasty medications

Table 3 shows the pharmacological management of the patient.

**Table 3:** Pharmacological management of the patient. mg: milligram, tab: tablet, Inj: injection, Pan: pantaprazole.

Medications	Dosage
Nebulization (Duolin and Budecort)	Thrice a day (eight hourly)
Tab. Clamitone 500 mg	Twice a day × two days
Inj. Pan40	Once a day × seven days
Inj. Hydrocort 500 mg	Once a day × five to seven day
Tab. Mucinac 600 mg	Thrice a day × seven days
Tab Angispan 2.5	Twice a day × five days
Tab Clopitab	Once a day × seven days

Therapeutic interventions

Counseling and education for both patients and caregivers to lower the risk of integumentary complications following surgery, to reduce post-operative respiratory and circulatory systems, to improve bed mobility and prevent prolonged immobility, to promote airway clearance and alleviate dyspnea, to increase lung volumes and capacities, to avoid stiffness in the joints and maintain their mobility and structural integrity, and to get back to routine activity of daily living (ADLs).

Physiotherapy management

Table 4 shows physiotherapy management from day one to day three. The patient was intubated via an endotracheal tube.

**Table 4:** Post-operative physiotherapy management from day one to day three.

Sr. no	Physiotherapy goals	Interventions	Dosage
1.	To promote his family member's awareness of the condition and obtain their consent.	Education and counseling for caregivers regarding the exercise program and the significance of following it.	Education was provided to caregivers regarding the significance of positioning every two hours, early mobility, and daily living activities.
2.	To reduce post-operative respiratory, circulatory system, and integumentary risks.	Positioning-air beds were provided; ankle-toe movements were given; a half-lying position was initially given; and it progressed to upright sitting.	The positioning was done every two hours. Initially, ten repetitions of a single set of ankle-to-toe movements were done twice a day; later, three to four times a day were added.
3.	To increase bed mobility and avoid being immobilized for too long.	Bedside mobilization and transitional training from rolling to side-lying, side-lying to sitting, and sitting to supported standing were given.	Rolling and side-lying for one to three days; sitting and supported standing for four to eight days
4.	To encourage clearing of the airways.	Mild chest vibrations; suctioning (oral and endotracheal tube)	Five oscillations x three sets twice a day.

Table 5 shows physiotherapy management from day three to week one. The patient was on nasal prongs.

**Table 5:** Post-operative physiotherapy management from day three to week one. post-op: post-operative, cc: cubic centimeter, ADL: activity of daily living.

Sr. no	Physiotherapy goal	Interventions	Dosage
1.	To relieve dyspnea	Breathing exercises: diaphragmatic breathing; pursed lip breathing.	Ten repetitions x one set twice a day at first, then ten repetitions x two sets
2.	To encourage the clearing of the airways	Coughing and huffing techniques (to remove secretions)	Ten repetitions x one set twice a day at first, then ten repetitions x two sets
3.	To overcome anxiety and depression	Relaxation techniques like Jacobson's relaxation	Five repetitions x one set
4.	To increase lung capacities and volumes	Exercises for thoracic expansion: fully flexed shoulder with deep inspiration and fully extended shoulder with expiration	Ten repetitions x one set twice a day at first, then ten repetitions x two sets three to four times daily.
		Initially, incentive spirometry was performed using a flow-oriented spirometer. Observational feedback via various balls that stand for 600, 900, and 1200cc	Beginning on the third day following surgery, at first two to three times a day, and then every two hours following that. On day three, <600cc without hold progressed upto >900cc with three seconds hold on day 13.
5.	To avoid joint stiffness and preserve joint mobility and integrity	Upper and lower extremities: active range of motion exercises.	Ten repetitions x one set twice a day at first, then ten repetitions x two sets three to four.
6.	Returning to regular ADLs	Walking at self-pace in a 10-meter hallway with assistance.	Initiate on post-op day four with 10 meters, gradually increasing to 30 meters.

**Figure 3:**
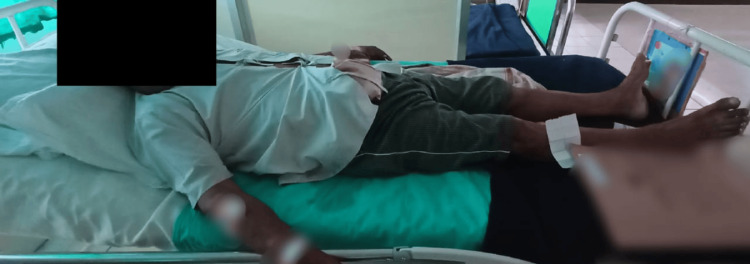
Jacobson's relaxation techniques to reduce anxiety and stress.

**Figure 4:**
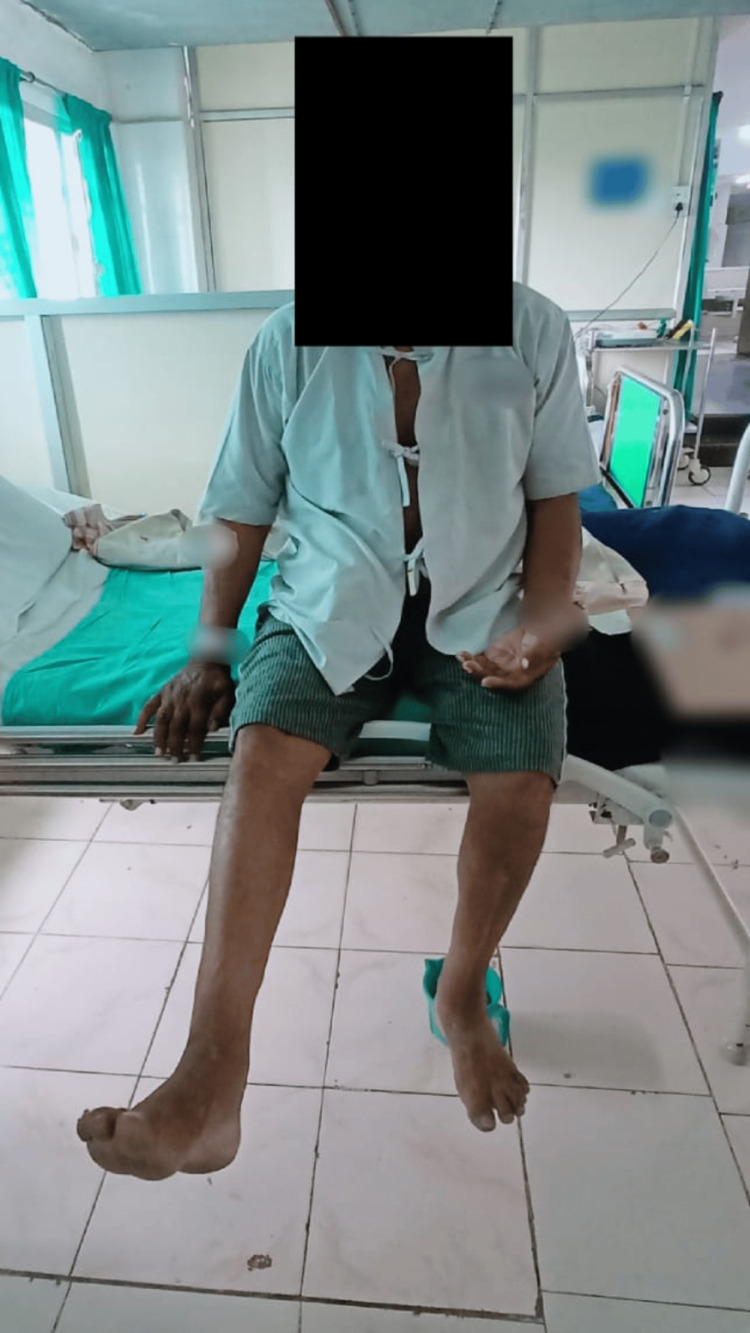
Dynamic quadriceps exercises.

**Figure 5:**
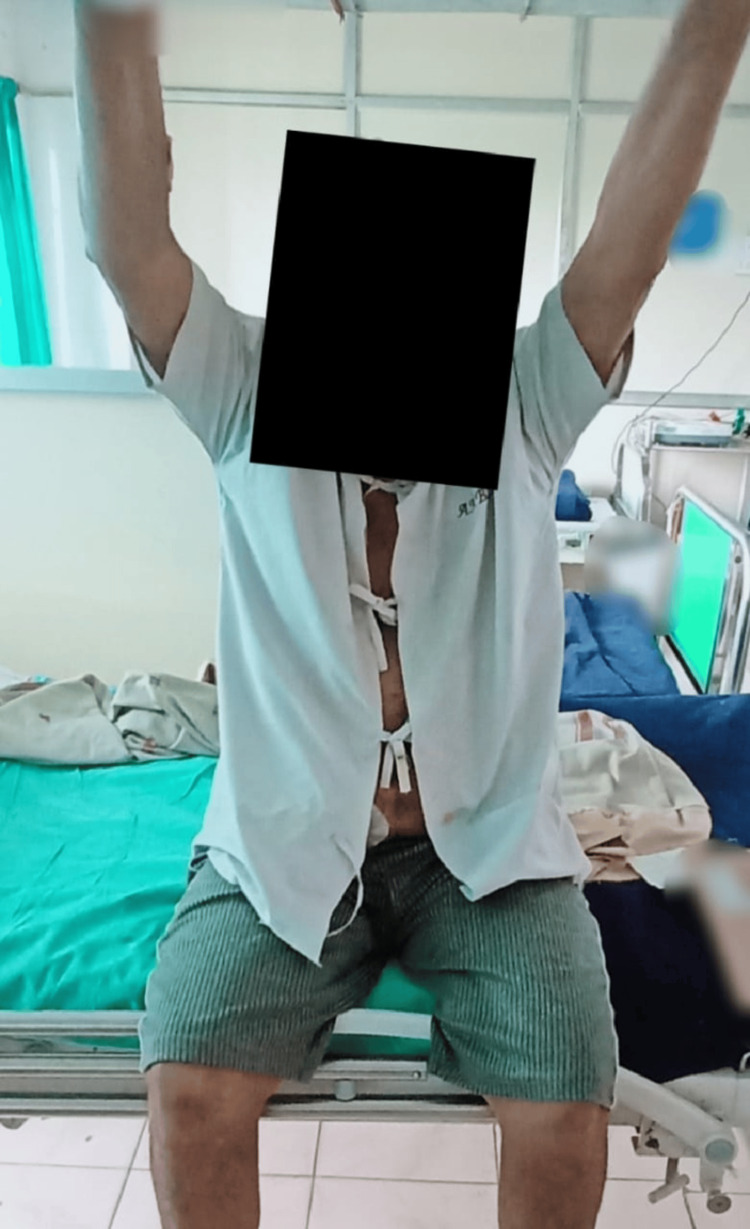
Thoracic expansion exercises with upper limb mobility.

Table 6 shows physiotherapy management from week one to week two. The patient was on room ventilation. Figure 3 shows Jacobson's relaxation techniques; Figure 4 shows dynamic quadriceps exercises; and Figure 5 shows thoracic expansion. All the exercises are performed by the patient, as mentioned in the figure.

**Table 6:** Post-operative physiotherapy management from week one to week two.

Sr. no	Physiotherapy goals	Interventions	Dosage
1.	To encourage airway clearance and removal of secretions	Active cycle of breathing technique	From the eighth day onwards. Ten repetitions x one set twice a day
2.	To increase chest expansion	Segmental breathing	Ten repetitions x one set twice a day at first, then ten repetitions x two sets three to four.
3.	To increase bed mobility and avoid being immobilized for too long	Standing unsupported, walking without assistance and spot marching.	For five minutes, progress to 10-15 minutes.
4.	To maintain muscle strength and endurance	Upper limb strengthening with a one-liter water bottle and lower limb strengthening with manual resistance.	Started one week after surgery. Ten repetitions x one strengthening; proceed to two sets per day.

Outcome measures

**Figure 6:**
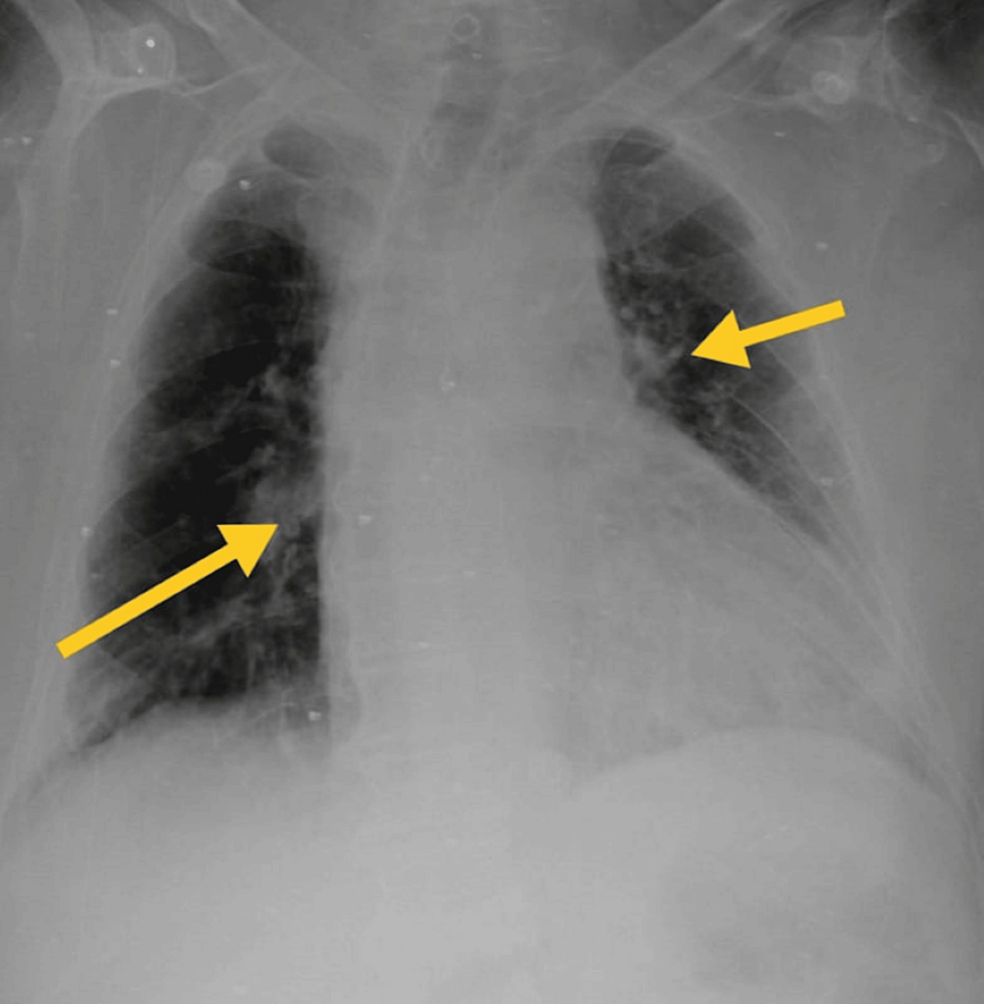
X-ray on 22nd October 2023. The yellow arrows show heterogeneous opacity, prominent bronchovascular marking, mucoid impaction in dilated bronchi, and branching opacities in the bilateral lungs.

**Figure 7:**
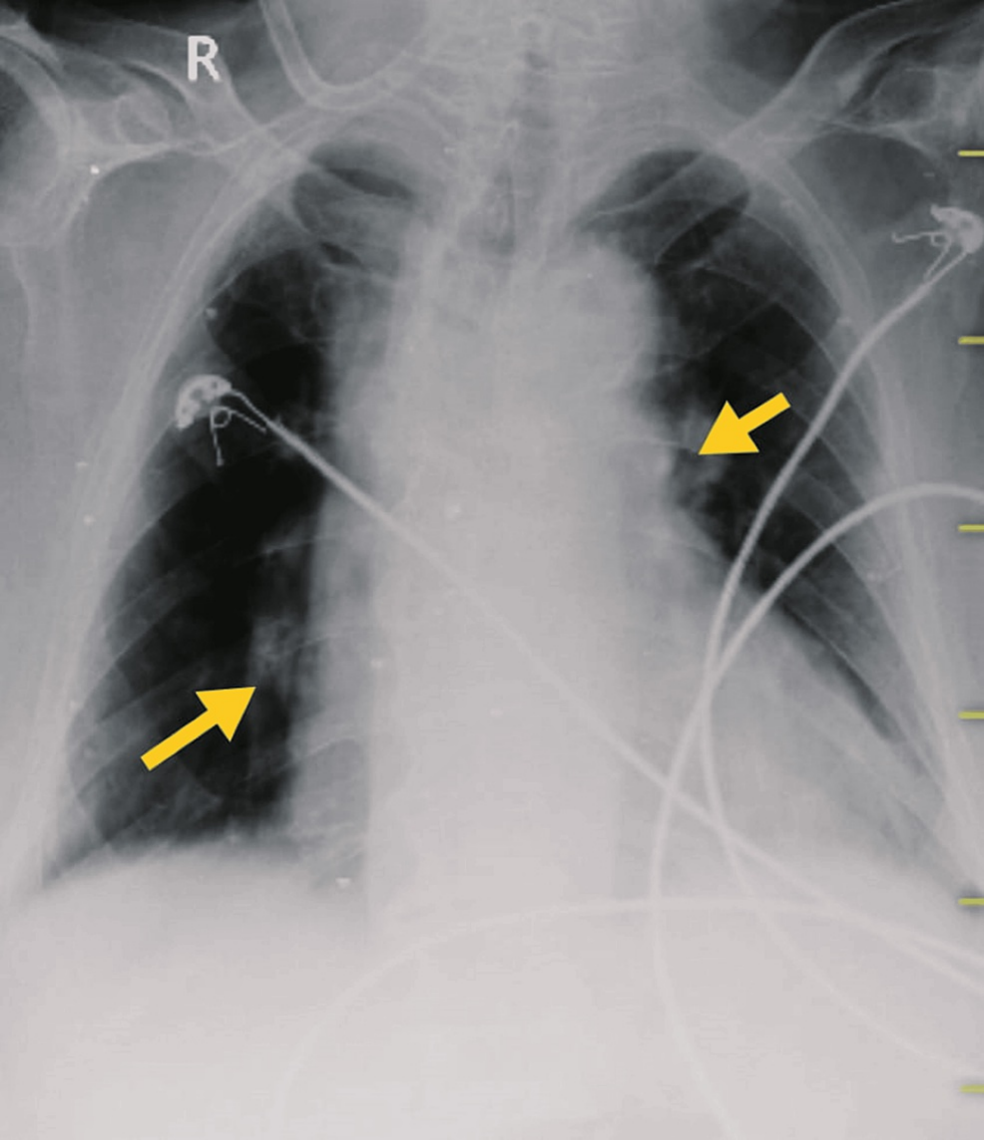
X-ray on 30th October 2023. The yellow arrows show mild mucoid impaction in the bronchi in the bilateral lungs.

ICU mobility scale [10], CURB-65 score for pneumonia severity [11], and X-ray grading scores [12] were used to assess patient progression. Tables 7, 8 show the outcome measures of the patient. In the inpatient department, every week, follow-up was taken to monitor the patient's condition. After discharge, the patient continued exercising at home and came back after two weeks for follow-up. Figures 2, 6, 7 show the X-ray, which is mentioned in Table 8.

**Table 7:** Outcome measures of a patient.

Outcome measure	Day one	Week one	Week two
ICU mobility scale	0/10	5/10	9/10
CURB-65 score for pneumonia severity	4/5	2/5	1/5
Chest X-ray grading scores	4/5	3/5	2/5

**Table 8:** Thorax radiological findings were used as the outcome.

On 14th October 2023 (day one)	On 22nd October 2023 (day seven)	On 30th October 2023 (Day 14)
Homogeneous opacity in the left middle and lower lung fields, cavitary lesion in the apical region, and obscured diaphragmatic outline and bilateral costo-phrenic angle in the left lung.	Heterogeneous opacity, prominent bronchovascular marking, mucoid impaction in dilated bronchi, branching opacities in bilateral lungs, and mild cardiomegaly.	Mild cardiomegaly with mild mucoid impaction in the bronchi.

## Discussion

A patient developed pneumonia after angioplasty, managed with proper medications and cardio-respiratory physiotherapy. The patient was intubated and referred for physical rehabilitation to minimize complications and resume daily activity. Intubated adults with pneumonia who are on mechanical ventilation may benefit from respiratory physiotherapy. Chest physiotherapy includes chest vibrations and suctioning initially [13-15]. Pattanshetty et al. proposed that chest physiotherapy is widely accepted as a beneficial adjuvant for airway clearance in mechanically ventilated patients with pneumonia or relapsing lung atelectasis, using body positioning, manual chest manipulation (vibration), and suctioning [14]. Achttien et al. suggest that patients' awareness of deep breathing and coughing exercises can prevent surgical complications, potentially leading to improved outcomes like reduced atelectasis and pneumonia [16]. Sadeghimoghaddam et al. suggest that relaxation and prayer therapy interventions can be beneficial for health in promoting hope and reducing anxiety in patients with CAD or pneumonia [17].

Moreover, physiotherapy protocol, which includes deep breathing exercises, incentive spirometry, and mobilization techniques, has been proven to enhance strength, mobility, range of motion, and fitness [18]. Monisha et al. suggest that active breathing techniques can improve coughing efficiency, clear secretions, and enhance ventilation by incorporating breathing exercises [19]. According to Kachpile et al., ICU mobility scores show a steady rise in mobility, indicating the effectiveness of physiotherapy interventions in improving patients' mobility. This improvement may be attributed to factors like physiotherapy, medical management, and the patient's response to treatment. Regular monitoring and adjustments to the physiotherapy plan may be necessary for ongoing rehabilitation [20]. So, we used this scale for ICU mobility scores to evaluate elderly patients' progress. The case report aims to expedite patient recovery by treating cardiovascular and respiratory complications. The patient took a two-week physical therapy program and continued the protocol up to four weeks, which significantly enhanced their condition, and they resumed daily activity.

## Conclusions

A case report highlighted the importance of a comprehensive physiotherapy approach in managing pneumonia after angioplasty in an elderly hypertensive patient. The physiotherapy approach was mainly focused on respiratory, circulatory, and psychological aspects and relaxation in relation to optimizing patient recovery. The majority of therapeutic objectives were met after two weeks of intensive physical therapy and continued up to four weeks. Significant improvements in the ICU mobility scale, CURB-65 score, and X-ray grading scores were also observed. A study emphasized the benefits of pulmonary rehabilitation programs to ensure an adequate and early recovery in a patient.
